# Conjunctival mass as an initial presentation of mantle cell lymphoma: a case report

**DOI:** 10.1186/1756-0500-5-671

**Published:** 2012-12-04

**Authors:** Mahsa Khanlari, Babak Bagheri, Reza Vojdani, Mohammad Mohammadianpanah, Shahram Paydar, Yahya Daneshbod

**Affiliations:** 1 Institute of Hematopathology, Dr Daneshbod Pathology Laboratory, Shiraz, Iran; 2 Institute of Ophthalmology, Dr. Bagheri clinic, Shiraz, Iran; 3 Institute of Hematology -Oncology, Nemazee Hospital, Shiraz, Iran; 4 Institute of Radiation- Oncology, Nemazee Hospital, Shiraz University of Medical Sciences, Shiraz, Iran; 5 Institute of Trauma and surgery, Rajaee Hospital, Shiraz University of Medical Sciences, Shiraz, Iran; 6 Dr Daneshbod Pathology Laboratory, Dept. of Hematopathology, Shiraz, 7134777118, Iran

**Keywords:** Lymphoma, Orbit, Conjunctiva, Mantle cell lymphoma, t(11;14), SOX 11

## Abstract

**Background:**

To describe a rare manifestation of mantle cell lymphoma (MCL) in conjunctiva, with clinical, hisologic, immunohistologic and genetic findings together with review of the Literature.

**Case presentation:**

Most ocular adnexal lymphomas are extranodal marginal zone B-cell lymphomas of mucosa-associated lymphoid tissue (MALT). A few cases of ocular adnexal mantle cell lymphomas have been reported in the literature. We present a case of mantle cell lymphoma presenting as right conjunctival mass of at least three months duration in a 64-year-old man. Histopathologic examination showed a proliferation of monomorphous small-to-medium-sized lymphoid cells with cleaved nuclei in the subconjunctiva. By immunohistochemistry, the infiltrate was positive for CD20, CD5, BCL-2, cyclin D1, and the transcription factor SOX11. Fluorescent in situ hybridization demonstrated the presence of *IGH-CCND1* fusion indicating t(11;14).

**Conclusion:**

A rigorous approach to initial diagnosis and staging of small cell lymphomas of the ocular adnexa is needed. The recognition of ocular MCL requires appropriate immunohistochemical staining and/or genetic confirmation to differentiate this rare form of presentation of MCL from other more frequent small cell lymphomas.

## Background

Ocular adnexal lymphomas (OALs) are the most common orbital neoplasms in adults, accounting for about 55% of all malignant orbital lesions [1,2]. About 80–90% of primary ocular adnexal lymphomas are extranodal marginal zone B-cell lymphomas of mucosa-associated lymphoid tissue (MALT), which can arise within the intraconal and extraconal orbital fat, lacrimal gland, extraocular muscles, lacrimal sac, lids or conjunctiva [3]. Mantle cell lymphoma (MCL) is a relatively rare lymphoma, accounting for less than 10% of all lymphomas. In the ocular adnexal region, including rare cases of conjunctiva, MCL accounts for between 1-5% [4] and 9% of the lymphomas [5]. The orbit is the most commonly involved site of MCL involvement, followed by the lacrimal gland and the eyelid, similar to extranodal marginal zone lymphoma in this region [6]. Mantle cell lymphomas presenting in the ocular adnexal region have a male predominance and are associated with extraorbital extension at the time of diagnosis, advanced stage disease, short median progression-free survival (PFS), and older-age at diagnosis [6,7]. In rare case reports, a higher frequency of these tumors fail to co-express CD5, rendering differential diagnosis between conjunctival mantle cell lymphoma from extranodal marginal zone B-cell lymphomas of MALT more difficult [8]. We here report a case of MCL of the conjunctiva, as the first clinical presentation of advanced-stage mantle cell lymphoma, with extraocular lymph node and bone marrow involvement and review the literature in this field.

## Case report

**Figure 1:**
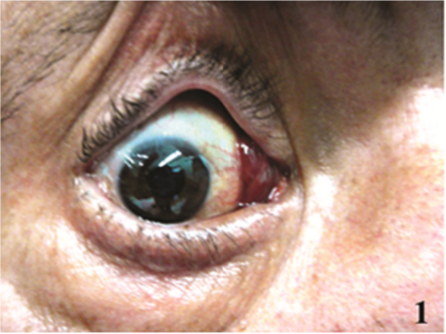
Right bulbar conjunctival mass, before treatment.

A 64-year-old man presented with a dark red palpable mass in the bulbar conjunctiva (diameter: 0.5 cm) at the medial canthus of the right eye. The mass was reported to be present for at least three months (Figure 1). The past medical history was uneventful and in particular no B-symptoms were reported. CT scans of the right orbit showed a limited bulbar conjunctival mass not involving the lacrimal gland or orbital septum of the eyelids.

**Figure 2:**
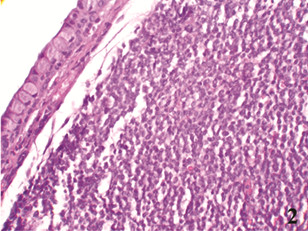
Histology of conjunctival mass biopsy showing monomorphous infiltration of cleaved lymphoid cells (H&E X20).

Histologic examination of an incisional biopsy showed infiltration by a monomorphic cell population composed of small-to-medium-sized lymphocytes with irregular, indented, or cleaved nuclei, 3/10 HPF mitotic rate and partially nodular growth pattern, beneath the simple cuboidal cell layer of surface conjunctival epithelium (Figure 2).

**Figure 3:**
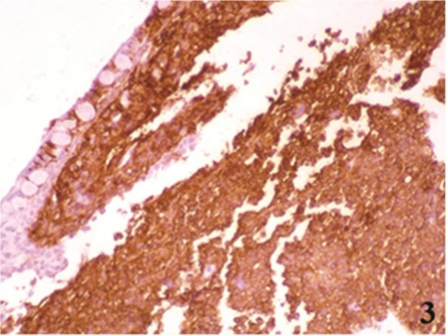
Immunohistochemistry study for CD20 (,X20).

**Figure 4:**
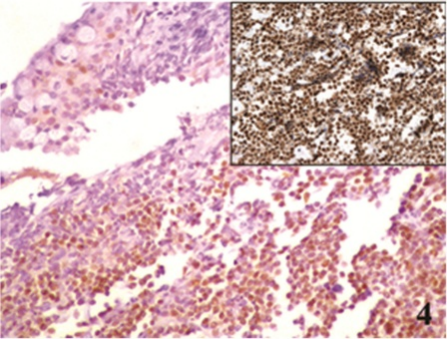
Immunohistochemistry study for Cyclin D1 (,X20), and (inset) Sox11 (,X20).

**Figure 5:**
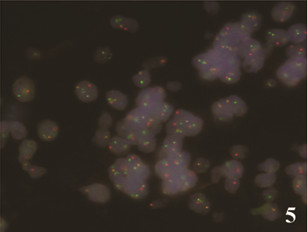
**FISH using the LSI IGH/CCND1 probe showing juxtaposition of IGH (green) and *CCND1 *(red) signals in the majority of cells indicating *IGH-CCND1 *rearrangement associated with t(11;14)(q13;q32).**

Immunohistochemical staining was performed on formalin-fixed, paraffin-embedded tissue sections, according to the manufacturers’ protocols. The primary antibodies used, the methods of antigen retrieval, and the dilution rates were as follows: CD20 (L26) (Novocastra, Newcastle, UK; microwave in 0.01 molar citrate buffer; 1:50), CD3 (PS1) (Novocastra, Newcastle, UK;microwave in 0.01 molar citrate buffer; 1:200), CD5 (4C7) (Novocastra, Newcastle, UK; microwave in RE7113 solution; 1:100), CD10 (56C6) (Novocastra, Newcastle, UK; microwave in citrate buffer; 1:100), CD23 (1B12) (Novocastra, Newcastle, UK; microwave in 0.01molar citrate buffer; 1:100), BCL-2 (3.1) (Novocastra, Newcastle, UK; microwave in citrate buffer; 1:100), cyclin D1 (P2D11F11) (Novocastra, Newcastle, UK; Trypsin digestion; 1:50), Ki-67 (MM1) (Novocastra, Newcastle, UK; microwave in citrate buffer; 1:100) and SOX11 (Atlas Antibodies, Stockholm, Sweden; heat-induced retrieval with ER2 BondMax buffer; 1:100) as previously described [9]. The tumor cells were positive for CD20 (Figure 3), CD5, BCL-2, cyclin D1 (Figure 4), and SOX-11 (Figure 4 inset) and negative for CD3, CD10, and CD23. The Ki-67 proliferation index was 12%. By fluorescence in situ hybridization (FISH) analysis on formalin-fixed, paraffin-embedded tissue sections using commercially available LSI IGH/CCND1 XT dual color, dual fusion and LSI CCND1 BAP break apart probes (Abbott-Vysis), juxtaposition of *IGH* and *CCND1* and a breakpoint in the *CCND1* locus were detected in 85% and 97% of nuclei, respectively, indicating presence of the MCL hallmark translocation t(11;14)(q13;q32) (Figure 5). The histopathologic and genetic findings confirmed the diagnosis of conjunctival mantle cell lymphoma.

**Figure 6:**
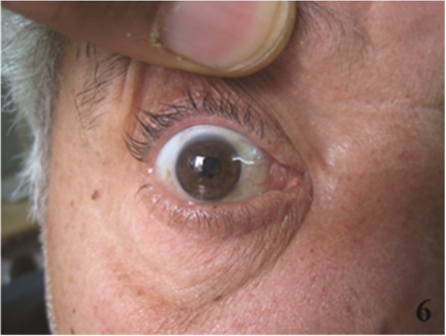
Right eye, 5 months after treatment.

The patient did not show up in his follow up Ophtholmology visit, however after two months was referred to an oncologist. Extensive clinical staging was performed and cervical lymph node and bone marrow involvements were discovered. Gastroscopy and laboratory workups such as liver function test, ESR, BUN, creatinine, β2 microglobulin, LDH, leukocyte count and serum protein electrophoresis were in normal ranges. The patient was allocated to the low-risk group with the MCL International Prognostic Index. Combined chemotherapy with Fludarabine, Cyclophosphamide and Rituximab (FCR) was administered. After five cycles (approximately 5 months) patient showed clinical response as a decrease in neck lymph-node sizes and shrinkage of the conjunctival lesion (Figure 6), however complete remission workup is pending after the sixth cycle.

## Consent

“Written informed consent was obtained from the patient for publication of this case report and any accompanying images. A copy of the written consent is available for review by the Editor-in-Chief of this journal.”

## Discussion

Ocular adnexal lymphomas (OAL) account for 2% of all non-Hodgkin lymphomas, and about 6-8% of extranodal lymphomas [2]. The primary involvement of the conjunctiva by lymphoma, comprises about one third of ocular adnexal lymphoma, and 1.5% of all conjunctival tumors. Like other primary ocular adnexal lymphomas, most of the primary conjunctival lymphomas are low-grade extranodal marginal zone B-cell lymphomas of MALT. Other types of lymphomas such as diffuse large-cell lymphoma, mantle cell lymphoma, and more aggressive histologic subtypes are less commonly seen [10]. It seems that, MCL in the ocular adnexal region is associated with poorer prognosis compared with the much more common extranodal marginal zone lymphoma of mucosa-associated lymphoid tissue type in the same region. Of the ocular adnexal MCL, 80% present in stage III/IV. The median overall survival has been reported to be only 57 months [7].

According to the largest published series (n=21) of ocular adnexal MCL including six cases with conjunctival localization, patients with involvement of the orbital and adnexal region as first presenting symptom (n=14) had more frequently bilateral eye and bone marrow involvement, and inferior OS as opposed to patients with secondary ocular adnexal MCL (n=7) [5]. The majority of patients in this series presented with stage IV disease with nodal- and bone-marrow involvement, although all patients were allocated in the low-risk group according to the MCL International Prognostic Index score.

In multiple studies, patients with lymphoma of conjunctival localization had a better outcome than lymphomas of other areas of the eye considering both local and systemic relapse [11]. Only 31% patients with conjunctival lymphoid tumors showed systemic lymphoma, with fewer progressions than those involving other areas of the orbit [1]. However, in recent studies conjunctival lymphomas with nodal involvement have a worse prognosis, and systemic disease is more common in patients with lymphomas located at an extralimbal site (ie, in the fornix or midbulbar region) [1,12]. A study analyzing prognostic factors exclusively for low grade conjunctival non-Hodgkin’s lymphoma identified four prognostic factors for lower disease-free survival (DFS) and overall survival (OS) in the univariate analysis, namely age greater than 59 years, nodal involvement, stage IV, and an elevated IPI, but only the nodal involvement maintained the negative impact on DFS in multivariate analysis [12].

OAL are a distinct form of clinical presentations of several types of lymphomas including MZL or MCL. Since the majority of patients with OALs initially present to ophthalmologists, the current subdivision of OALs to primary versus secondary adnexal lymphomas, based on limited eye involvement (by imaging) and evidence of disease dissemination or previous history of lymphoma, is not practical, and the frequency of systemic dissemination at the time of diagnosis in patients who present with initial ocular complaints is largely unknown [13]. In line with this, our case although had initially presented with occular lymphoma limited to conjunctiva, but due to inadequate workup by ophthalmologist and loosing follow up, subdividing our case to primary or secondary was not possible. Our case expressed nuclear SOX11 and cyclin-D1, as most classical MCLs. SOX11 negative MCLs are mainly leukemic and have a non-nodal presentation. Although nodal dissemination in SOX11 negative patients may occur and they are usually associated with *TP53* mutation protein expression and more aggressive behavior [14].

## Conclusion

In conclusion, a rigorous approach to initial diagnosis and staging of small cell lymphomas of the ocular adnexa is needed. The recognition of ocular MCL requires appropriate immunohistochemical staining and/or genetic confirmation to differentiate this rare form of presentation of MCL from other more frequent small cell lymphomas. Also according to TNM staging of ocular lymphomas, orbital imaging as well as systemic workup is suggested for these rare ocular presentations.

## Competing interest

No conflict of interest is declared, and source of funding is Dr Daneshbod Laboratory.

## Authors’ contributions

"BB" made clinical diagnosis, "MK"and "YD" carried out the diagnostic histology and immunohistologic studies and drafted the article, "RV", "MM" and "SP" participated in the treatment and revising the manuscript. All authors read and approved the final manuscript.
